# Methodology and application of PCR‐RFLP for species identification in tuna sashimi

**DOI:** 10.1002/fsn3.1552

**Published:** 2020-06-07

**Authors:** Lin Yao, Jianping Lu, Meng Qu, Yanhua Jiang, Fengling Li, Yingying Guo, Lianzhu Wang, Yuxiu Zhai

**Affiliations:** ^1^ Key Laboratory of Testing and Evaluation for Aquatic Product Safety and Quality Ministry of Agriculture and Rural Affairs Qingdao China; ^2^ Yellow Sea Fisheries Research Institute Chinese Academy of Fishery Sciences Qingdao China; ^3^ College of Food Science and Engineering Ocean University of China Qingdao China

**Keywords:** Cyt *b*, PCR‐RFLP, sashimi, species identification, tuna

## Abstract

The Thunnini, or tuna, comprise many species with very different commercial values. The principal raw tuna product on the market is sashimi, for which the species used is difficult to identify through conventional morphological analysis. The present study amplified the cytochrome *b* gene (*Cytb*) of 4 major tuna species used for preparing sashimi—yellowfin tuna (*Thunnus albacares*), southern bluefin tuna (*Thunnus maccoyii*), bigeye tuna (*Thunnus obesus*), and Atlantic bluefin tuna (*Thunnus thynnus*)—and 4 species commonly mislabeled as components of tuna sashimi—albacore tuna (*Thunnus alalunga*), skipjack tuna (*Katsuwonus pelamis*), striped marlin (*Tetrapturus audax*), and swordfish (*Xiphias gladius*). Polymerase chain reaction (PCR) amplicons were digested with 5 restriction enzymes—*Eco*147 I, *Hinf* I, *Mbo* I, *Xag* I, and *Hind* II—to obtain characteristic restriction maps of the above‐mentioned raw tuna species and the commonly mislabeled species. An identification method using PCR restriction fragment length polymorphism (PCR‐RFLP) was established and validated using 39 commercial tuna sashimi samples, which verified that this method provides results consistent with those obtained by classical sequencing. PCR‐RFLP has several advantages over classical sequencing, such as simplicity, speed and accuracy. This technique could support species identification for raw tuna and sashimi.

## Graphical abstract

Find out the restriction fragment length polymorphism in the Cyt *b* gene of most common kinds of tuna for sashimi preparation. Established a simply and accurate method for most common kinds of tuna identification. This method provides the technology needed to support the rapid identification of raw tuna species for quality inspections and scientific research.

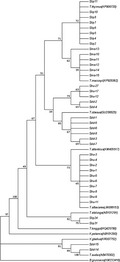

## 1 INTRODUCTION

Tuna is one of the 3 major types of fish recommended by the International Union of Nutritional Sciences for its high nutritional value. The Thunnus and Katsuwonus genera, including the Atlantic bluefin tuna (*Thunnus thynnus*), southern bluefin tuna (*Thunnus maccoyii*), yellowfin tuna (*Thunnus albacares*), bigeye tuna (*Thunnus obesus*), albacore tuna (*Thunnus alalunga*), and skipjack tuna (*Katsuwonus pelamis*), are generally of great economic value (FAO, 2010). Atlantic and southern bluefin, yellowfin, and bigeye tuna are high‐end seafood products primarily sold in the form of sashimi and sushi (Kurokura, Takagi, Sakai, & Yagi, 2012). These species account for more than half of all tuna production. The commercial value of different tuna species varies widely, leading to fraudulent seafood mislabeling in both domestic and international markets. For example, fresh striped marlin (*Tetrapturus audax*), swordfish (*Xiphias gladius*), and albacore tuna are often mislabeled, while yellowfin and bigeye tuna are mislabeled in sushi (Lowenstein, Amato, & Kolokotronis, 2009). In addition, between 60% and 94% of the fish sold as red snapper in the United States are mislabeled (Marko et al., 2004). Seafood mislabeling circumvents consumer choice, which poses health risks, impacts the normal business order of the market, and undermines conservation strategies. Therefore, it is necessary to establish a method to identify the species of raw tuna used to produce sashimi.

Methods for species identification primarily include conventional morphological identification, protein‐based analysis, and molecular biology techniques based on PCR. Morphological identification relies on the specialist's knowledge of systematic taxonomy and long‐term experience, but it is difficult to differentiate similar species with close genetic relationships using this approach. Protein‐based analyses, exemplified by isozyme analysis and immunological analysis, still lack stability and specificity. Therefore, molecular biology techniques are currently more widely used for species identification in research (Aranishi, Okimoto, & Izumi, 2005; Calo‐Mata et al., 2009). This includes random amplified polymorphic DNA (RAPD), amplified fragment length polymorphism (AFLP), and forensically informative nucleotide sequencing (FINS). However, these methods also exhibit some limitations, such as complex procedures, poor stability, and poor reproducibility (Chapela et al., 2007; Larraín, Díaz, Lamas, Uribe, & Araneda, 2014; Rasmussen & Morrissey, 2009). The polymerase chain reaction‐restriction fragment length polymorphism (PCR‐RFLP) technique, which was developed based on fingerprinting technology, is most widely used for species identification because of its simple procedure, low cost, and good reproducibility (Sivaraman et al., 2018; Wilwet, Jeyasekaran, Shakila, Sivaraman, & Padmavathy, 2018). Moreover, it just needs PCR thermocycler and electrophoresis apparatus, which means most laboratories could bear the cost, that is also a realistic factor in most developing countries.

The present study investigated 4 major tuna species for sashimi preparation (yellowfin, southern and Atlantic bluefin, and bigeye tuna) and 4 commonly mislabeled species (albacore and skipjack tuna, striped marlin, and swordfish). A method for identifying raw tuna species was established using PCR amplification of the cytochrome *b* gene (*Cytb*) and restriction enzyme digestion, and was validated in commercial samples. This method provides a novel approach for the rapid and simple identification of raw tuna species.

## 2 MATERIALS AND METHODS

### 2.1 Samples

Eight kinds of species samples including albacore tuna (cm), yellowfin tuna (hu), Atlantic bluefin tuna (lq), southern bluefin tuna (ms), bigeye tuna (dd), striped marlin (da), swordfish (ji), and skipjack tuna (jy) from main fishing ocean were collected by Shandong ZhongLu Oceanic Fisheries Co., Ltd. Each kind of species had 20 muscle samples from 20 individual fish, which were morphologically identified by expert. The 39 commercial sashimi products labeled as yellowfin tuna (Shu), southern bluefin tuna (Sms), bigeye tuna (Sdd), and Atlantic bluefin tuna (Slq) were purchased randomly from 4 major supermarkets and 4 sushi restaurants with different suppliers and batches in Qingdao, China.

### 2.2 Instruments and reagents

TIANGEN Marine Animal Tissue DNA Extraction Kit and 2× Taq PCR MasterMix were purchased from TIANGEN BIOTECH Co., Ltd. The DNA marker was purchased from Solarbio Science & Technology Co., Ltd. The restriction enzymes *Eco*147 I, *Hinf* I, *Mbo* I, *Xag* I, and *Hind* II were purchased from Takara Biomedical Technology Co., Ltd. Primer synthesis and sequencing were performed by Sangon Biotech Co., Ltd. The PCR machine used was the traditional T1 PCR instrument (Whatman Biometra). The gel imaging system used was INFINITY 3000 (Vilber Lourmat Sté). Spectrophotometer used was NanoPhotometer Pearl (Implen LLC, German).

### 2.3 DNA extraction

Thirty milligrams of fish flesh was cut into pieces and placed in a centrifuge tube. DNA extraction was performed according to the manufacturer's protocol. The DNA concentration and purity were measured using the NanoPhotometer Pearl. The samples were stored at −20°C.

### 2.4 PCR amplification of the Cytb gene

The primers used in this study were *Cytb*‐1:5'‐CCATCCAACATCTCAGCATGATGAAA‐3' and *Cytb*‐2:5'‐CCCTCAGAATGATATTTGTCCTCA‐3'. The length of the amplified fragment was approximately 350 bp (Marko et al., 2004). The PCR system is shown in Table 1.

**Table 1:** PCR system of Cyt *b* gene

Reagent	Concentration	Sampling volume
PCR MasterMix	2×	25 μl
Cyt *b*‐1	10.0 μM	1.0 μl
Cyt *b*‐2	10.0 μM	1.0 μl
DNA	~100 ng/μl	1.0 μl
ddH_2_O	–	22.0 μl

Conditions for the PCR were as follows: pre‐denaturation at 95°C for 10 min; 35 cycles of 94°C for 1 min, 53°C for 1 min, 72°C for 1 min; and extension at 72°C for 10 min.

The PCR amplicons were subjected to electrophoresis on a 1.0% agarose gel at 120 V for 40 min. The experimental results were observed and recorded using a gel imaging system. The PCR amplicons were sequenced by Sangon Biotech Co., Ltd.

### 2.5 Sequence alignment and phylogenetic tree analysis

Following manual correction, the sequences of the 8 fish species were aligned using the Blast online tool (http://blast.ncbi.nlm.nih.gov/). The *Cytb* gene of yak (*Bos grunniens*; GeneBank: KM233416) was used as the out‐group, and the *Cytb* genes of 8 tuna species (*Thunnus alalunga* AB101291, *Thunnus albacares* JN086153, *Thunnus maccoyii* KF925362, *Thunnus obesus* GU256525, *Thunnus thynnus* KF906720, *Katsuwonus pelamis* AB101290, Thunnus tonggo HQ425780, and *Thunnus atlanticus* KM405517) and 2 deep‐sea fish species (*Tetrapturus audax* AB470302 and *Xiphias gladius* KR007752) were selected from the NCBI Web site as reference genes. A phylogenetic tree was constructed using the MEGA 7 software (https://www.megasoftware.net) with 1,000 bootstrap replicates. The Kimura 2‐parameter was used for modeling, and neighbor‐joining was used to construct the phylogenetic tree.

### 2.6 Restriction enzyme analysis

The cleavage sites of the *Cytb* PCR amplicons were analyzed using DNAMAN (https://www.lynnon.com). Five species‐specific restriction enzymes were selected and used to digest the PCR amplicons of the *Cytb* gene: *Eco*147 I, *Hinf* I, *Mbo* I, *Xag* I, and *Hind* II (for identification of samples dd and jy only).

The PCR amplicons (10.0 μl) were mixed with 1.0 μl of the respective restriction enzymes, 2.0 μl of 10× FastDigest^®^Buffer, and 17.0 μl of deionized water. After enzyme digestion at 37°C for 60 min, the results were analyzed using agarose gel electrophoresis on a 3.0% gel.

## 3 RESULTS

### 3.1 PCR amplification

**Figure 1:**
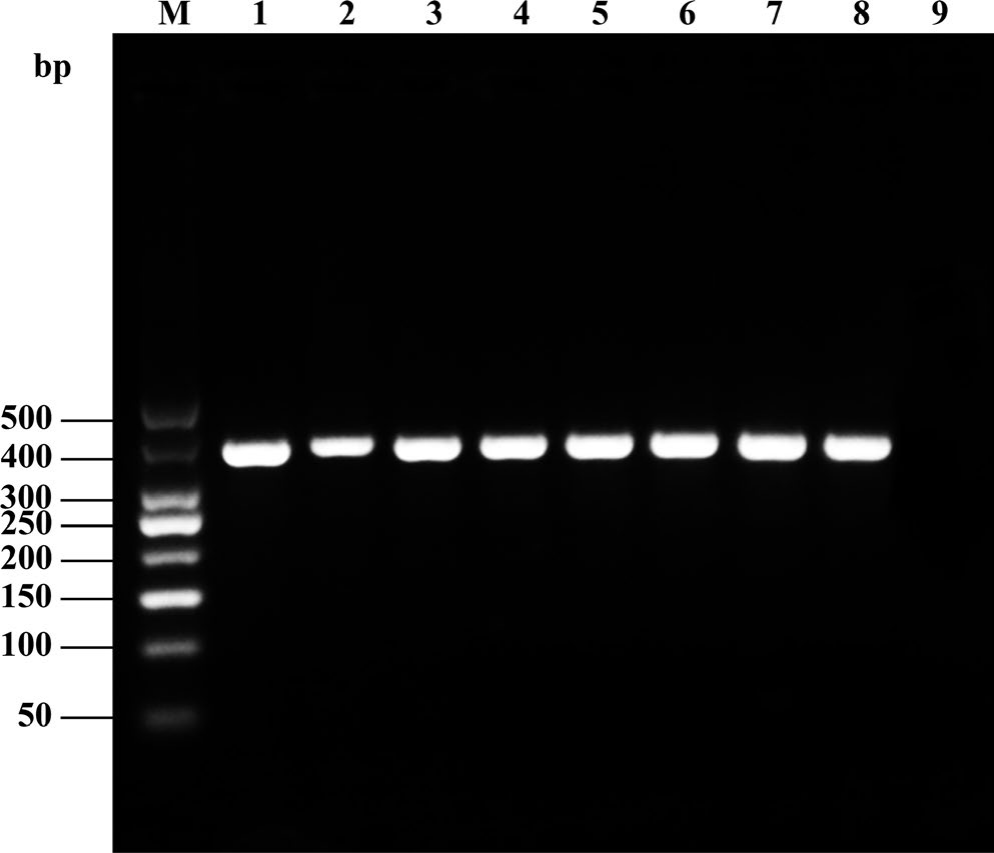
Electrophoresis of Cyt *b* gene PCR products in eight species of fish

A single fragment was amplified from each experimental sample. No fragment length polymorphism was detected. All fragments had a length of 357 bp (Figure 1), as expected.

### 3.2 Sequence alignment and phylogenetic tree analysis

**Figure 2:**
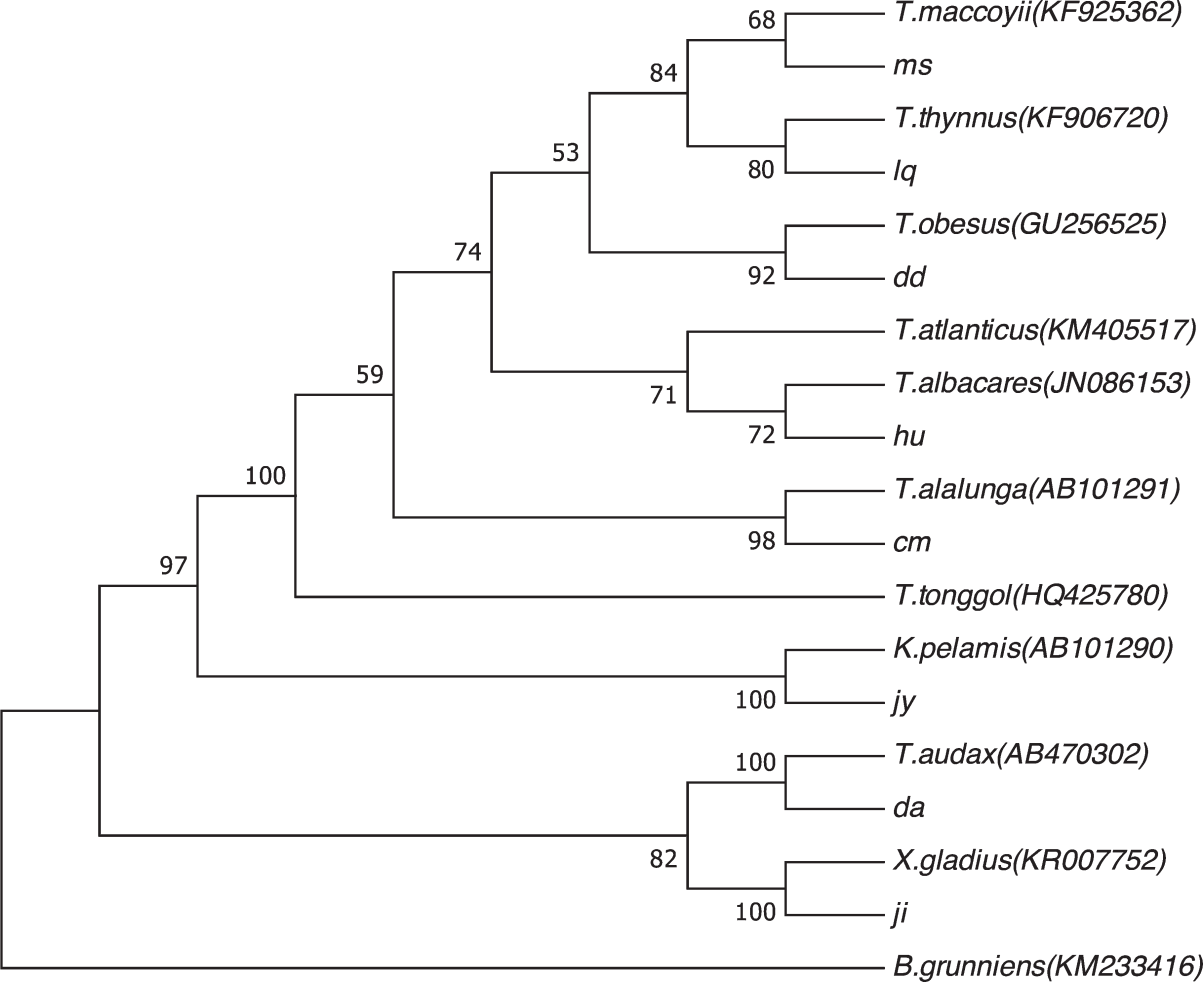
Eight species of fish Cyt *b* gene phylogenetic tree

The sequencing products were aligned against the GenBank database to determine the *Cytb* region in the mitochondrial DNA (mtDNA). The phylogenetic tree constructed based on the *Cytb* gene of the 8 fish species is shown in Figure 2. Each of the following sample/species pairs clustered together in a single branch: the southern bluefin tuna sample (ms) and *Thunnus maccoyii*; the Atlantic bluefin tuna sample (lq) and *Thunnus thynnus*; the bigeye tuna sample (dd) and *Thunnus obesus*; the yellowfin tuna sample (hu) and *Thunnus albacares*; the albacore tuna sample (cm) and *Thunnus alalunga*; the skipjack tuna (jy) and *Katsuwonus pelamis*; the striped marlin sample (da) and *Tetrapturus audax*; and the swordfish sample (ji) and *Xiphias gladius*. The phylogenetic analysis shows that the result of molecular identifies was in accordance with the morphological identification completely, confirming the authenticity of 8 species samples used in this study.

### 3.3 Results and analysis of enzymatic digestion of the PCR amplicons

#### 3.3.1 Albacore tuna (Thunnus alalunga)

**Figure 3:**
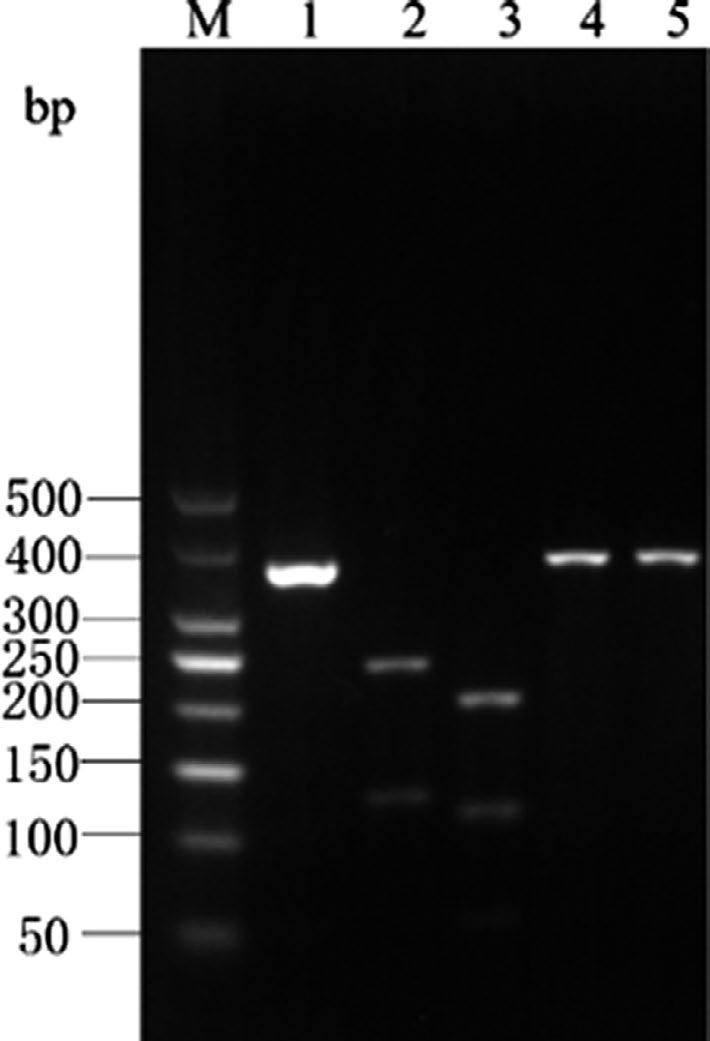
Enzyme digestion result of Cyt *b* gene PCR product in *T. alalunga*

The *Cytb* PCR amplicon of albacore tuna was digested by *Eco*147 I into 2 bands of 233‐ and 124‐bp lengths, and by *Hinf* I into 3 bands of 109‐, 52‐, and 196‐bp lengths. There were no restriction sites for *Mbo* I and *Xag* I in the *Cytb* PCR amplicon of albacore tuna; hence, a single band of 357‐bp length was obtained upon digestion by these enzymes. The enzyme digestion results for albacore tuna are shown in Figure 3 and are consistent with the predictions made using the DNAMAN software.

#### 3.3.2 Yellowfin tuna (Thunnus albacare)

**Figure 4:**
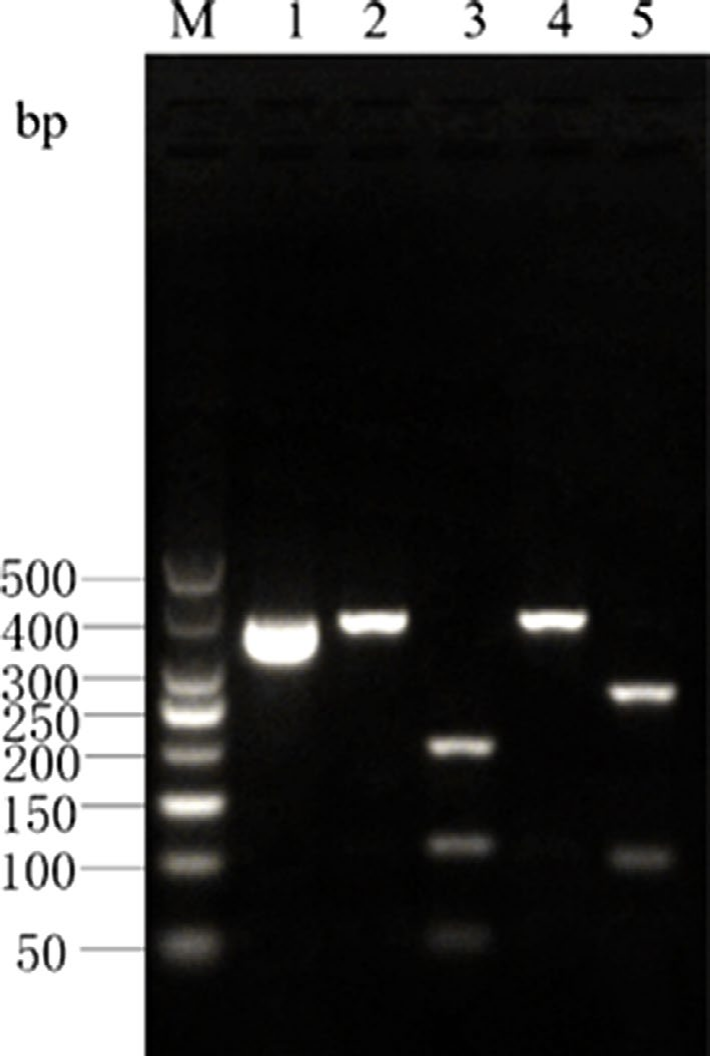
Enzyme digestion result of Cyt *b* gene PCR product in *T. albacares*

The *Cytb* PCR amplicon of yellowfin tuna was digested by *Hinf* I into 3 bands of 109‐, 52‐, and 96‐bp lengths, and by *Xag* I into 2 bands of 101‐ and 256‐bp lengths. There were no restriction sites for *Eco147* I and *Mbo* I in the *Cytb* PCR amplicon of yellowfin tuna; hence, a single band of 357‐bp length was obtained upon digestion by these enzymes. The enzyme digestion results for yellowfin tuna are shown in Figure 4 and are consistent with the predictions made using the DNAMAN software.

#### 3.3.3 Atlantic bluefin tuna (Thunnus thynnus)

**Figure 5:**
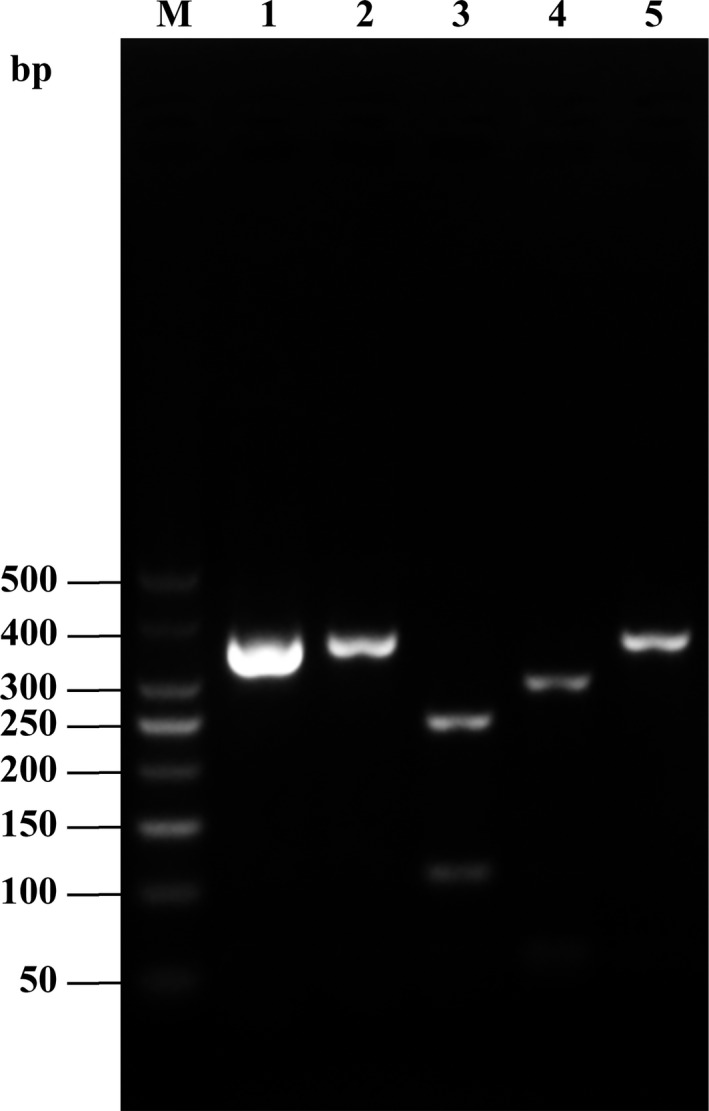
Enzyme digestion result of Cyt *b* gene PCR product in *T.thynnus*

The *Cytb* PCR amplicon of Atlantic bluefin tuna was digested by *Hinf* I into 2 bands of 109‐ and 248‐bp lengths, and by *Mbo* I into 2 bands of 62‐ and 295‐bp lengths. There were no restriction sites for *Eco147* I and *Xag* I in the *Cytb* PCR amplicon of Atlantic bluefin tuna; hence, a single band of 357‐bp length was obtained upon digestion by these enzymes. The enzyme digestion results for Atlantic bluefin tuna are shown in Figure 5 and are consistent with the predictions made using the DNAMAN software.

#### 3.3.4 Southern bluefin tuna (Thunnus maccoyii)

**Figure 6:**
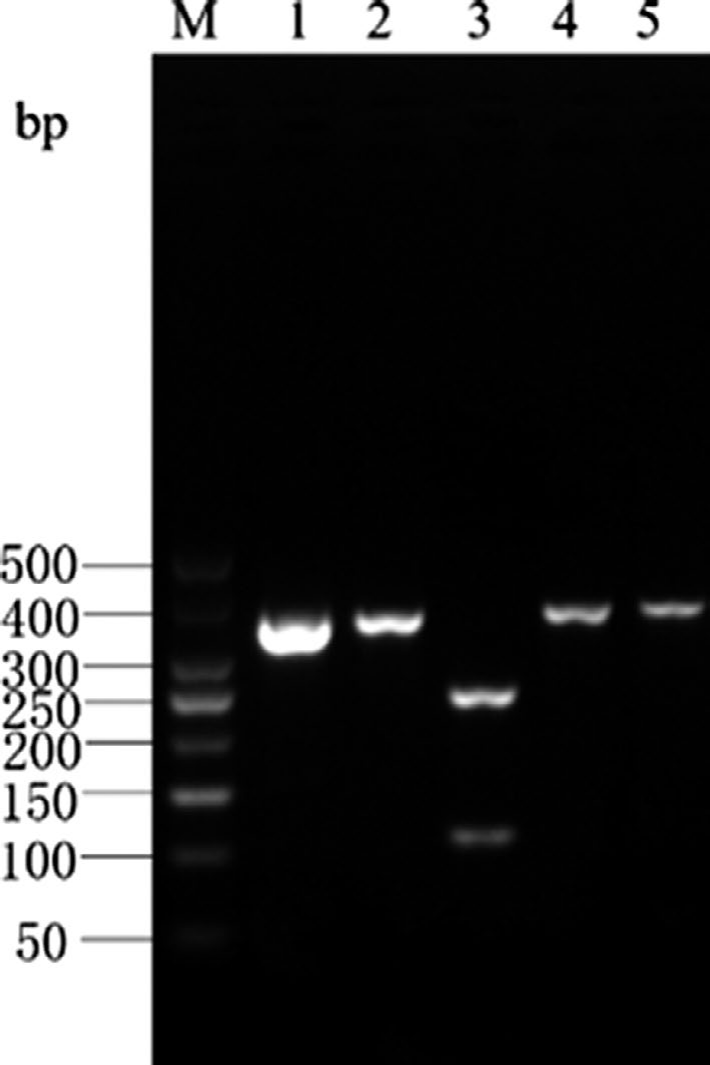
Enzyme digestion result of Cyt *b* gene PCR product in *T. maccoyii*

The *Cytb* PCR amplicon of southern bluefin tuna was digested by *Hinf* I into 2 bands of 109‐ and 248‐bp lengths. There were no restriction sites for *Mbo* I, *Eco147* I, and *Xag* I in the *Cytb* PCR amplicon of southern bluefin tuna; hence, a single band of 357‐bp length was obtained upon digestion by these enzymes. The enzyme digestion results for southern bluefin tuna are shown in Figure 6 and are consistent with the predictions made using the DNAMAN software.

#### 3.3.5 Striped marlin (Tetrapturus audax)

**Figure 7:**
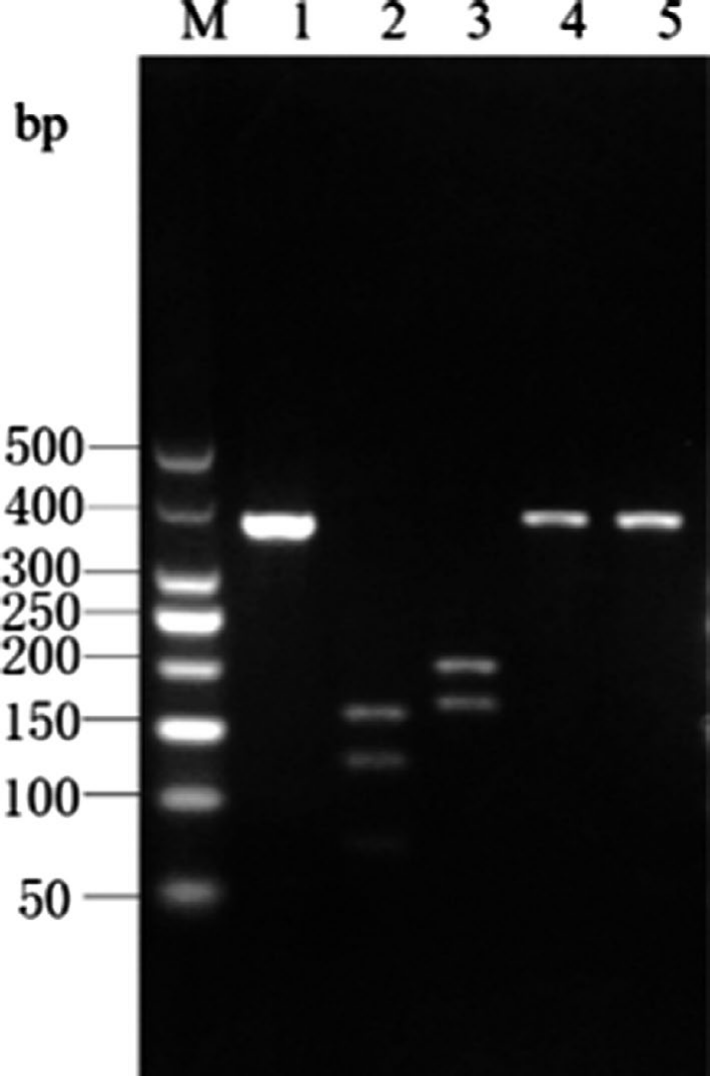
Enzyme digestion result of Cyt *b* gene PCR product in *T. audax*

The *Cytb* PCR amplicon of striped marlin was digested by *Eco147* I into 3 bands of 124‐, 74‐, and 159‐bp lengths, and by *Hinf* I into 2 bands of 196‐ and 161‐bp lengths. There were no restriction sites for *Mbo* I and *Xag* I in the *Cytb* PCR amplicon of striped marlin; hence, a single band of 357‐bp length was obtained upon digestion by these enzymes. The enzyme digestion results for striped marlin are shown in Figure 7 and are consistent with the predictions made by the DNAMAN software.

#### 3.3.6 Swordfish (Xiphias gladius)

**Figure 8:**
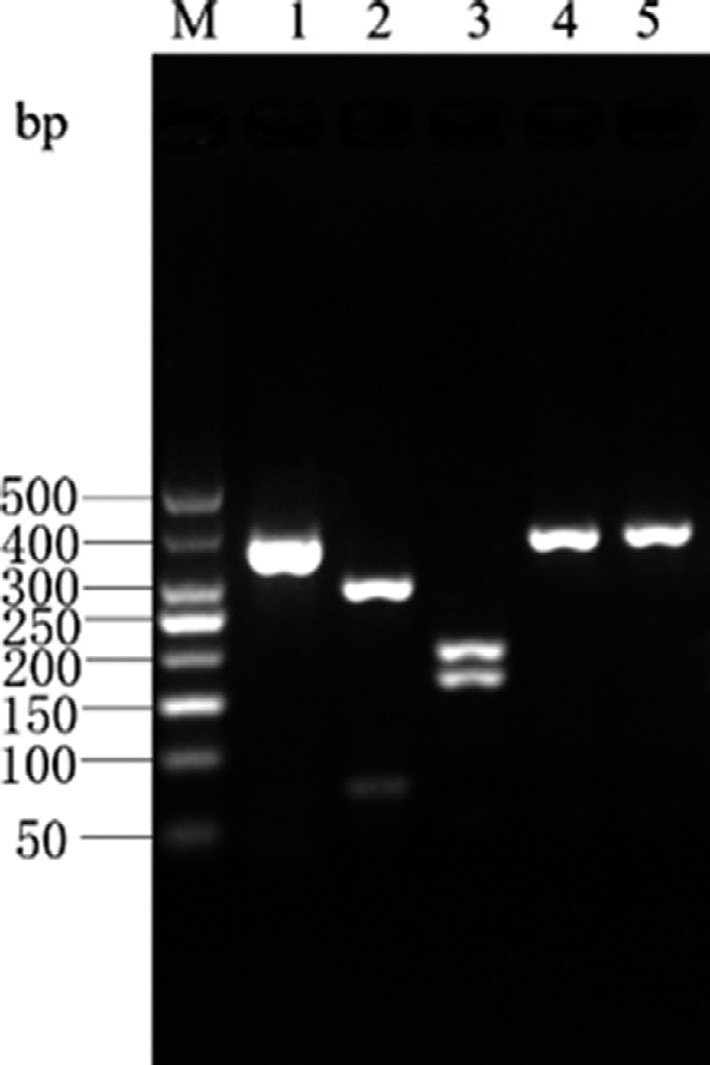
Enzyme digestion result of Cyt *b* gene PCR product in *X. gladius*

The *Cytb* PCR amplicon of swordfish was digested by *Eco147* I into 2 bands of 283‐ and 74‐bp lengths, and by *Hinf* I into 2 bands of 196‐ and 161‐bp lengths. There were no restriction sites for *Mbo* I and *Xag* I in the *Cytb* PCR amplicon of swordfish; hence, a single band of 357‐bp length was obtained upon digestion by these enzymes. The enzyme digestion results for swordfish are shown in Figure 8 and are consistent with the predictions made by the DNAMAN software.

#### 3.3.7 Bigeye tuna (Thunnus obesus)

**Figure 9:**
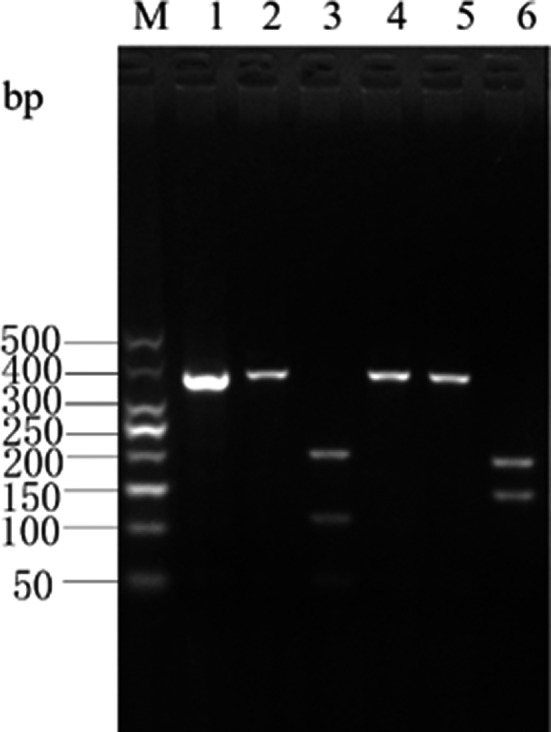
Enzyme digestion result of Cyt *b* gene PCR product in *T. obesus*

The *Cytb* PCR amplicon of bigeye tuna was digested by *Hinf* I into 3 bands of 109‐, 52‐, and 196‐bp lengths, and by *Hind* II into 3 bands of 150‐, 10‐, and 197‐bp lengths. There were no restriction sites for *Eco147* I, *Mbo* I, and *Xag* I in the *Cytb* PCR amplicon of bigeye tuna; hence, a single band of 357‐bp length was obtained upon digestion by these enzymes. The enzyme digestion results for bigeye tuna are shown in Figure 9 and are consistent with the predictions made by the DNAMAN software.

#### 3.3.8 Skipjack tuna (Katsuwonus pelamis)

**Figure 10:**
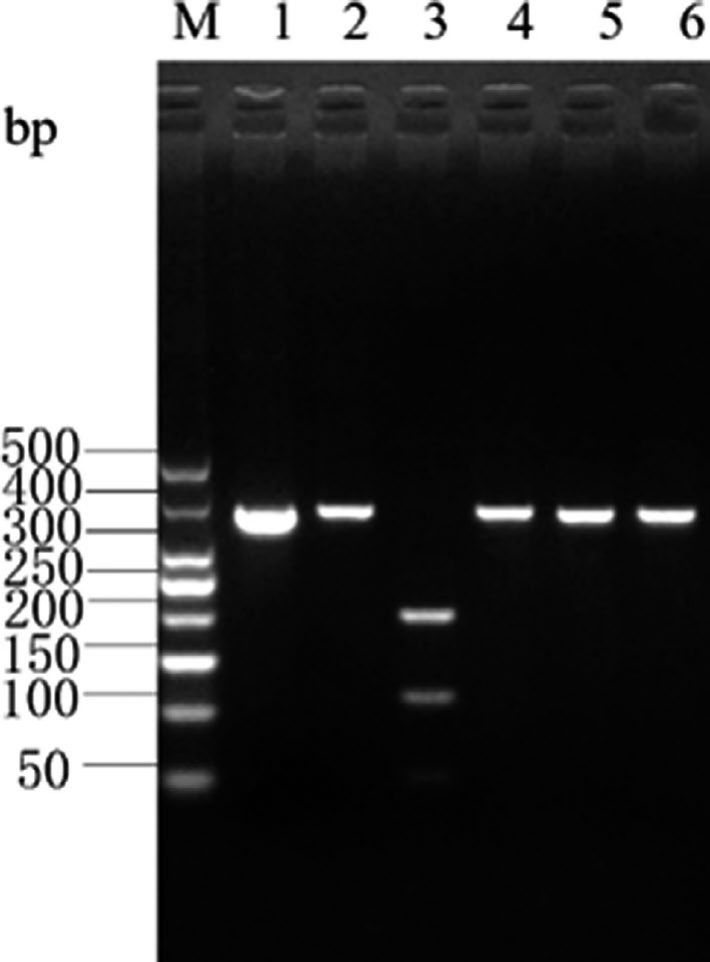
Enzyme digestion result of Cyt *b* gene PCR product in *K. pelamis*

The *Cytb* PCR amplicon of skipjack tuna was digested by *Hinf* I into 3 bands of 109‐, 52‐, and 196‐bp lengths. There were no restriction sites for *Eco147* I, *Mbo* I, *Xag* I, and *Hind* II in the *Cytb* PCR amplicon of skipjack tuna; hence, a single band of 357‐bp length was obtained upon digestion by these enzymes. The enzyme digestion results for skipjack tuna are shown in Figure 10 and are consistent with the predictions made by the DNAMAN software.

The results of restriction enzyme analysis of the *Cytb* gene PCR amplicons of the 8 fish species are shown in Table 2.

**Table 2:** Statistics of enzyme digestion results for 8 kinds of fish of Cyt *b* gene PCR product

Number	Species	Restriction enzyme				
		*Eco147* I	*Hinf* I	*Mbo* I	*Xag* I	*Hind* II
cm	*T. alalunga*	233, 124	109, 52, 196	357	357	–
hu	*T. albacares*	357	109, 52, 196	357	101, 256	–
lq	*T. thynnus*	357	109, 248	62,295	357	–
ms	*T. maccoyii*	357	109, 248	357	357	–
da	*T. audax*	124, 74, 159	196, 161	357	357	–
ji	*X. gladius*	283, 74	196, 161	357	357	–
dd	*T. obesus*	357	109, 52, 196	357	357	150, 10, 197
jy	*K. pelamis*	357	109, 52, 196	357	357	357

#### 3.3.9 Results of commercial sample analysis

**Figure 11:**
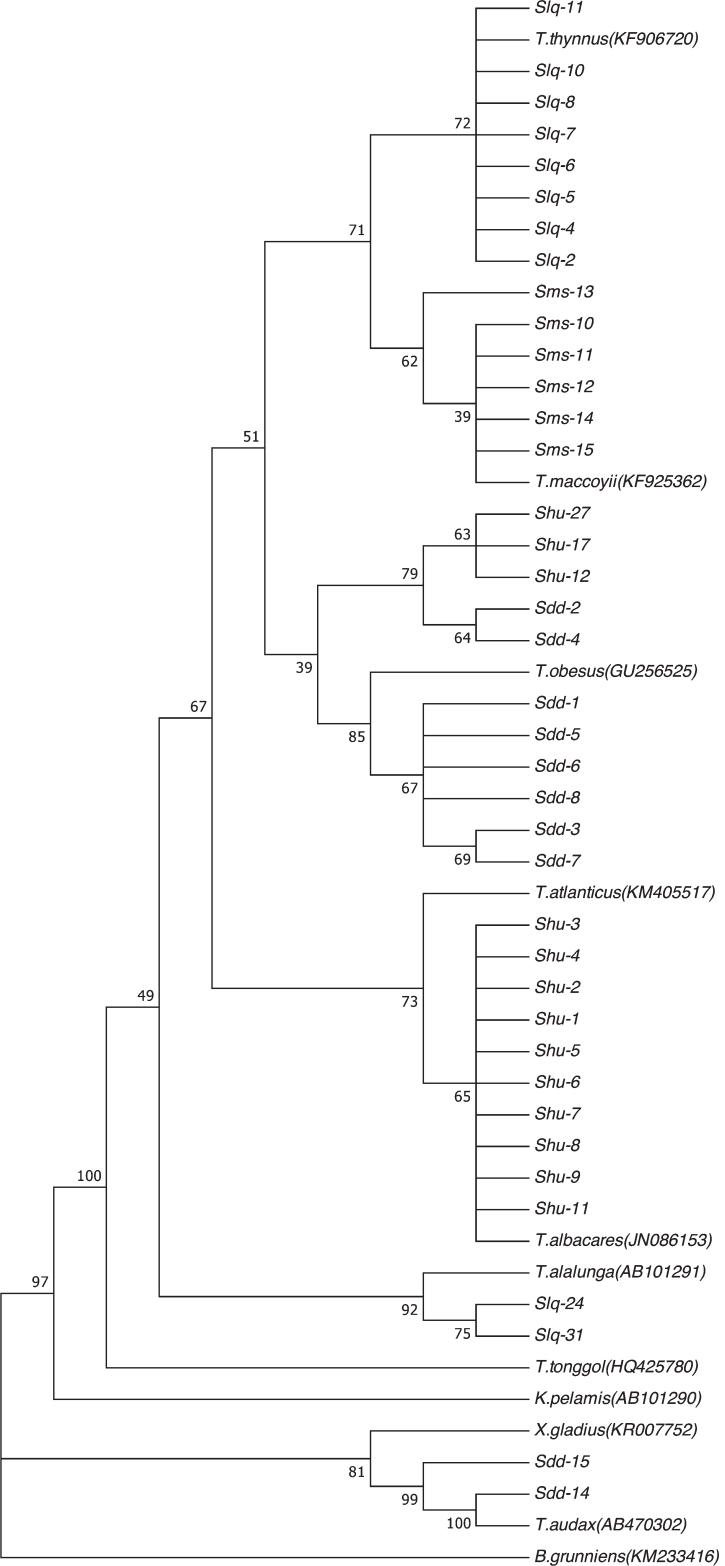
Phylogenetic tree of Cyt *b* gene in 39 sample fishes on market

After the method of identifying raw tuna by PCR‐RFLP was established, this study identified the species of 39 commercial tuna samples and compared these results against the phylogenetic tree constructed based on *Cytb* gene sequences of the 39 commercial samples (shown in Figure 11). Of the 10 samples labeled as Atlantic bluefin tuna, the PCR‐RFLP results identified 8 samples as Atlantic bluefin tuna and the other 2 samples as albacore tuna, which is consistent with the sequence alignment results and phylogenetic tree analysis. Out of the 6 samples labeled as southern bluefin tuna, the PCR‐RFLP results identified all samples as southern bluefin tuna, which was consistent with the results of the sequence alignment and phylogenetic tree analysis. Of the 13 samples labeled as yellowfin tuna, the PCR‐RFLP results identified 10 samples as yellowfin tuna and the other 3 samples as bigeye tuna which was consistent with the results obtained using sequence alignment and phylogenetic tree analysis. Of the 10 samples labeled as bigeye tuna, the PCR‐RFLP results identified 8 samples as bigeye tuna and the other 2 samples as striped marlin, which was consistent with the sequence alignment results and phylogenetic tree analysis. Detailed results of the identification of the commercial samples are shown in Table 3.

**Table 3:** Commercial sample sequence analysis and PCR‐RFLP identification results

Sample number	Sample nominal	Sequence analysis	PCR‐RFLP identification
Slq‐2	Atlantic bluefin tuna	*Thunnus thynnus*	*Thunnus thynnus*
Slq‐4	Atlantic bluefin tuna	*Thunnus thynnus*	*Thunnus thynnus*
Slq‐5	Atlantic bluefin tuna	*Thunnus thynnus*	*Thunnus thynnus*
Slq‐6	Atlantic bluefin tuna	*Thunnus thynnus*	*Thunnus thynnus*
Slq‐7	Atlantic bluefin tuna	*Thunnus thynnus*	*Thunnus thynnus*
Slq‐8	Atlantic bluefin tuna	*Thunnus thynnus*	*Thunnus thynnus*
Slq‐10	Atlantic bluefin tuna	*Thunnus thynnus*	*Thunnus thynnus*
Slq‐11	Atlantic bluefin tuna	*Thunnus thynnus*	*Thunnus thynnus*
Slq‐24^*^	Atlantic bluefin tuna	*Thunnus alalunga*	*Thunnus alalunga*
Slq‐31^*^	Atlantic bluefin tuna	*Thunnus alalunga*	*Thunnus alalunga*
Sms‐10	Southern bluefin tuna	*Thunnus maccoyii*	*Thunnus maccoyii*
Sms‐11	Southern bluefin tuna	*Thunnus maccoyii*	*Thunnus maccoyii*
Sms‐12	Southern bluefin tuna	*Thunnus maccoyii*	*Thunnus maccoyii*
Sms‐13	Southern bluefin tuna	*Thunnus maccoyii*	*Thunnus maccoyii*
Sms‐14	Southern bluefin tuna	*Thunnus maccoyii*	*Thunnus maccoyii*
Sms‐15	Southern bluefin tuna	*Thunnus maccoyii*	*Thunnus maccoyii*
Shu‐1	Yellowfin tuna	*Thunnus albacares*	*Thunnus albacares*
Shu‐2	Yellowfin tuna	*Thunnus albacares*	*Thunnus albacares*
Shu‐3	Yellowfin tuna	*Thunnus albacares*	*Thunnus albacares*
Shu‐4	Yellowfin tuna	*Thunnus albacares*	*Thunnus albacares*
Shu‐5	Yellowfin tuna	*Thunnus albacares*	*Thunnus albacares*
Shu‐6	Yellowfin tuna	*Thunnus albacares*	*Thunnus albacares*
Shu‐7	Yellowfin tuna	*Thunnus albacares*	*Thunnus albacares*
Shu‐8	Yellowfin tuna	*Thunnus albacares*	*Thunnus albacares*
Shu‐9	Yellowfin tuna	*Thunnus albacares*	*Thunnus albacares*
Shu‐11	Yellowfin tuna	*Thunnus albacares*	*Thunnus albacares*
Shu‐12^*^	Yellowfin tuna	*Thunnus obesus*	*Thunnus obesus*
Shu‐17^*^	Yellowfin tuna	*Thunnus obesus*	*Thunnus obesus*
Shu‐27^*^	Yellowfin tuna	*Thunnus obesus*	*Thunnus obesus*
Sdd‐1	Bigeye tuna	*Thunnus obesus*	*Thunnus obesus*
Sdd‐2	Bigeye tuna	*Thunnus obesus*	*Thunnus obesus*
Sdd‐3	Bigeye tuna	*Thunnus obesus*	*Thunnus obesus*
Sdd‐4	Bigeye tuna	*Thunnus obesus*	*Thunnus obesus*
Sdd‐5	Bigeye tuna	*Thunnus obesus*	*Thunnus obesus*
Sdd‐6	Bigeye tuna	*Thunnus obesus*	*Thunnus obesus*
Sdd‐7	Bigeye tuna	*Thunnus obesus*	*Thunnus obesus*
Sdd‐8	Bigeye tuna	*Thunnus obesus*	*Thunnus obesus*
Sdd‐14^*^	Bigeye tuna	*Tetrapturus audax*	*Tetrapturus audax*
Sdd‐15^*^	Bigeye tuna	*Tetrapturus audax*	*Tetrapturus audax*

Note

"*" sample nominal is inconsistent with the result of identification

## 4 DISCUSSION

The rapid development of modern molecular biotechnology has led to an evolution in research approaches for species identification ranging from morphological, physiological, and biochemical levels, to the molecular level. The basis for identification has also evolved from the shape, size, color, bone, and structure of the fish, to macrobiomolecules, such as DNA, RNA, and proteins (Liu, Xu, Wu, Xie, & Feng, 2016). DNA‐based molecular identification approaches have been widely used for the identification of species commonly mislabeled as tuna. Liu et al. (2016) successfully identified 5 tuna species (southern bluefin, bigeye, yellowfin, albacore, and skipjack tuna) using real‐time fluorescent PCR. Lin and Hwang (2008) established a multiplex PCR assay to distinguish 5 small‐sized tuna species, including *Euthynnus pelamis* (also known as skipjack tuna), *Auxis rochei* (bullet tuna), *Auxis thazard* (frigate tuna), *Sarda orientalis* (striped bonito), and *Euthynnus affinis* (mackerel tuna). Abdullah and Rehbein (2016) utilized single‐strand conformation polymorphism to distinguish bullet tuna from the *Thunnus* genus. Vinas and Tudela (2009) successfully identified 8 tuna species (albacore, yellowfin, southern bluefin, bigeye, Atlantic bluefin, blackfin, longtail, and Pacific bluefin tuna) using the mitochondrial (mtDNA) control region gene and the Internal Transcribed Spacer‐1 (*ITS*1) gene. Puncher et al. (2015) utilized the cytochrome c oxidase subunit I (*CO*I) and *ITS*1 genes to successfully identify the larvae of Atlantic bluefin tuna collected from 3 spawning areas in the Mediterranean. Lowenstein et al. (2009) used DNA barcoding technology to identify 68 tuna sushi samples purchased from restaurants in Manhattan and found that the labels of 22 samples did not match the identified species. These techniques and approaches are relatively accurate at identifying the species of tuna and its products, but they also have several limitations, including complex operational procedures, lengthy identification times, or expensive equipment.

PCR‐RFLP has been successfully applied for the identification of a variety of fish species and their products due to its simplicity and low cost. Wolf, Hübner, and Lüthy (1999) identified 8 species of carp using PCR‐RFLP. Chakraborty, Aranishi, and Iwatsuki (2005) developed a PCR‐RFLP approach that could successfully identify 3 closely related hairtail species (genus *Trichiurus*). Akasaki, Yanagimoto, Yamakami, Tomonaga, and Sato (2006) found that PCR‐RFLP could rapidly identify 9 species of cod. Espiñeira, González‐Lavín, Vieites, and Santaclara (2008) used PCR‐RFLP to distinguish 7 species of anglerfish. Chen et al. (2012) successfully identified 5 species of pufferfish using PCR‐RFLP analysis and a chip bioanalysis system. Lin and Hwang (2007) utilized PCR‐RFLP, wherein 2 sets of primers were designed to partially amplify 126‐ and 146‐bp sequences of the *Cytb* gene and 5 restriction enzymes were used for digestion. They successfully identified the species in 18 commercial canned tuna products and could distinguish albacore, yellowfin, bigeye, and Atlantic bluefin tuna*,* but not southern bluefin tuna, which is mainly consumed raw.

In this study, PCR amplification of the *Cytb* gene was performed on 4 major sashimi tuna species (yellowfin, Atlantic and southern bluefin, and bigeye tuna) and 4 commonly mislabeled species (albacore and skipjack tuna, striped marlin, and swordfish). Five restriction enzymes, including *Eco147* I, *Hinf* I, *Mbo* I, *Xag* I, and *Hind* II, were used to digest the *Cytb* sequences of the 8 species to establish restriction maps specific for each species. Compared with the Lin and Hwang (2007) study, the current study placed more emphasis on the identification of fish species used for sashimi. In addition, only 1 pair of universal *Cytb* primers was used to successfully distinguish 6 tuna species, including southern bluefin tuna and 2 deep‐sea fish species, striped marlin and swordfish. This study also identified the species in 39 samples of commercial tuna sashimi by PCR‐RFLP and verified the results through phylogenetic tree analysis. The results showed that the species identified by PCR‐RFLP concurred with the sequencing and phylogenetic tree analysis results, demonstrating the accuracy of the method. The procedures required for PCR‐RFLP are simple and rapid, enabling the accurate identification of raw tuna and mislabeled species in a short time. Thus, this method provides the technology needed to support the rapid identification of raw tuna species for quality inspections and scientific research.

## CONFLICT OF INTEREST

The authors declare that they do not have any conflict of interest.
